# The genus *Trichocnemis* LeConte, 1851 (Coleoptera, Cerambycidae, Prioninae)

**DOI:** 10.3897/zookeys.61.299

**Published:** 2010-10-11

**Authors:** Ian Swift, Antonio Santos-Silva, Eugenio H. Nearns

**Affiliations:** 1 California State Collection of Arthropods, 3294 Meadowview Road, Sacramento, California 95832 USA; 2 Museu de Zoologia, Universidade de São Paulo, CP 188, 90001-970, São Paulo, SP, Brazil; 3 Department of Biology, Museum of Southwestern Biology, University of New Mexico, 167 Castetter Hall, MSC03 2020, Albuquerque, NM 87131-0001, USA

**Keywords:** Cerambycidae, Coleoptera, North American Fauna, Prioninae, taxonomy

## Abstract

The history of the genus Trichocnemis LeConte, 1851 (Coleoptera, Cerambycidae, Prioninae) is discussed. Its taxonomic status in relation to the genera Ergates Audinet-Serville, 1832 and Callergates Lameere, 1904 is clarified. The synonymy of Macrotoma californica White, 1853, Macrotoma spiculigera White, 1853, and Trichocnemis spiculatus LeConte, 1851 is confirmed. A key to all three genera and their species is provided.

## Introduction

The prionine genus Trichocnemis has not been formally recognized in North America since it was placed in synonymy with Ergates by Linsley (1962). While the two genera share several characters, and are likely related (Nishio 1956), many characters distinguish the species in these two genera. Earlier authors (Lameere 1901, Casey 1912) considered Trichocnemis a subgenus of Ergates, as was Callergates. More recent authors consider all three as separate genera (Villiers 1978, Jeniš 2001).

Members of all three genera are mainly Holartic in distribution: Ergates occurs in Europe and NW Africa (Jeniš 2008); Callergates occurs in Europe and Asia Minor (Jeniš 2001); and Trichocnemis occurs in western North America (Linsley 1962). Two species of Trichocnemis are recognized: Trichocnemis spiculatus LeConte, 1851 and Trichocnemis pauper Linsley, 1957. The species Trichocnemis spiculatus also has a single subspecies, Trichocnemis spiculatus neomexicanus Casey, 1890. Most species worldwide utilize coniferous hosts, generally in the genus Pinus (Pinaceae) (Linsley op.cit., Villiers 1978); however, Trichocnemis pauper is known only from species in the genus Quercus (Fagaceae) (Tyson 1967). Typically, recently dead host material is preferred by adult females for oviposition, and larval development ranges from two to four years (Linsley op.cit.), depending upon host and environmental conditions.

Males and females are strongly sexually dimorphic, with males having an enlarged, generally smooth prothorax with less distinct lateral spines, while in females the prothorax is smaller and more distinctly spined at the lateral margins. Adults are frequently attracted to ultraviolet lights at night, and are generally active during July and August (Tyson 1967).

## Methods

We examined the external morphology of male and female specimens of Trichocnemis spiculatus spiculatus, Trichocnemis spiculatus neomexicanus, Trichocnemis pauper, Ergates faber (Linnaeus, 1761), and Callergates gaillardoti (Chevrolat, 1854), in addition to male genitalia of one species of each genera as well as both species of Trichocnemis, to obtain the conclusions proposed in this study.

Specimens from the following collections were examined for this study:

BMNHThe British Museum of Natural History, London, United Kingdom

CASCCalifornia Academy of Sciences, San Francisco, California, USA

CSCACalifornia State Collection of Arthropods, Sacramento, California, USA

IRSNInstitute Royal des Sciences Naturelles de Belgique, Bruxelles, Belgium

INIAInstituto Nacional de Investigación y Tecnología Agraria y Alimentaria, Spain

EMEC	University of California Berkeley, Berkeley, CA USAabb

USNM	United States National Museum, Washington DC, USAabb

## Taxonomic History

**Figures 1–6.**
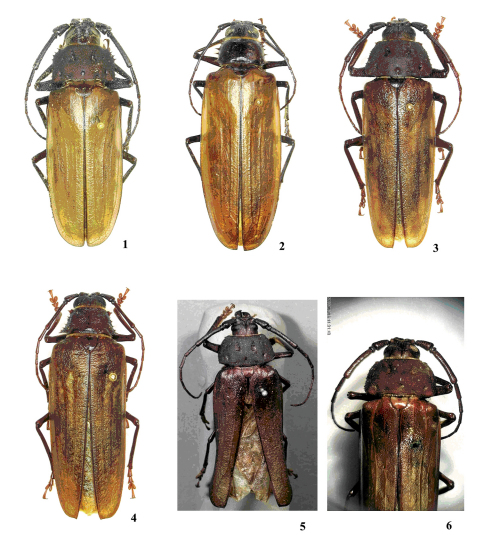
**1** Trichocnemis pauper, male **2** idem, female **3** Trichocnemis spiculatus neomexicanus, male **4** idem, female **5** Trichocnemis spiculatus spiculatus, holotype male (MCZWeb) **6** Macrotoma californica, syntype 1.

LeConte (1851) described the genus Trichocnemis for his new species Trichocnemis spiculatus, stating it was similar to the genus Ergates Audinet-Serville, 1832, but differing in the pubescence of the protibiae. Although LeConte did not indicate the sex of the type specimen, his description suggests it was a female: “Tibiae vix compresse, filiformes”; “thorace scabro, dorso antice bicalloso, spiculis lateralibus valde acutis, apicali basalique majoribus, thorace latioribus”; “the elytra show some indications of costae”; and “the joints of the antennae are marked with a few scattered punctures.” However, the type specimen deposited in the Museum of Comparative Zoology (MCZ) is a male (MCZWeb 2009). There is little doubt that LeConte (op. cit.) based his description on a single specimen, since he indicated only a single measurement, stating: “the specimen appears a little immature.” In males, the tibiae are not clearly filiform, the prothorax is not scabrous and has well-marked depressions (a character omitted by LeConte), the pronotal callosities are less pronounced than in females, the lateral spines of the pronotum are much less prominent than in females, and the proximal antennomeres are strongly and abundantly punctate. In the type specimen (Fig. 1), the elytra show clear carinae, a character that does not agree with the original description.

Although the holotype label of Trichocnemis spiculuatus in the MCZ indicates “Ergates”, and not Trichocnemis, it is believed that LeConte himself changed the label after having transferred the species to the genus Ergates. This is consistent with other LeConte specimens in which the labels indicate different names that the original taxon, for example: Mallodon gnatho LeConte, 1858, which have labels with LeConte’s writing, [Mallodon (Nothopleurus) gnatho // Lec. *dentiger* Lec.]. Other specimen labels are clearly not written by LeConte (*vide* Mallodon mandibularis Gemm.).

The genus Ergates was established by Audinet-Serville (1832) to accommodate a single species: Prionus serrarius Panzer, 1793 (= Cerambyx faber Linnaeus, 1761). Among the many characters used to define the genus, Audinet-Serville (op.cit.) listed: legs without internal spines; antennae filiform, similar in both sexes, longer than the body in male, and reaching more or less the middle of elytra in female; antennomere III longer than IV-V together; prothorax finely crenulated laterally in male, more distinctly in female; mandibles and mentum glabrous; legs of medium length, the prolegs longer than the others.

White (1853) synonymyzed Trichocnemis under Macrotoma Audinet-Serville, 1832, but this nomenclatural act was not accepted or used by any later author. White (op.cit.) also did not explain why, in his opinion, that genus was synonymous of Macrotoma.

LeConte (1854) then synonymyzed Trichocnemis with Ergates, stating: “Trichocnemis Lec. (Journ. Acad. 2d, 2, 110) is not sufficiently distinct from Ergates; the Californian species must therefore be called Ergates spiculatus.” Later, LeConte and Horn (1883) pointed out the same observation of Trichocnemis and placed Ergates in the tribe Ergatini. However, the characters used to define Ergatini (sensu LeConte and Horn) apply primarily to Ergates (= Trichocnemis) spiculatus, and largely excludes Ergates faber (Linnaeus, 1761) (Fig. 2) and Callergates gaillardoti (Chevrolat, 1854) (Fig. 3). LeConte and Horn (op.cit.) stated: “the tribe is easily recognized by the prothorax being much broader in the male than in the female, and finely punctured; in the latter sex the sculpturing is very coarse, and the small teeth of the lateral margin are longer and more acute. The head is small, the eyes reniform and coarsely granulated; antennae 11-jointed, slender, two-thirds the length of the body in the ♂, about half the length of the body in the ♀, rough with elevated punctures, with the 3rd joint as long as the three following united; poriferous spaces on the 3rd joint small inconspicuous, on the under surface near the distal end, gradually becoming larger, until the outer joints become entirely poriferous, and irregularly reticulated with fine elevated lines forming elongate cells, which are much less distinct, and in fact hardly to be seen in the male.” This tribal description excludes Ergates faber (Fig. 2) because the head is somewhat large, especially in males; the antennae surpass the elytral apex in males; antennomere III is (at most) as long as IV-V together; the pronotum lacks lateral spines in both sexes, and is not clearly wider in males than in females. Callergates gaillardoti can be unsatisfactorily included, because the antennae is somewhat longer than two-thirds the length of the body in male, the antennomere III is shorter than IV-VI together in both sexes, and the teeth of the lateral margin are not “small” in the female.

Lacordaire (1869) did not revalidate Trichocnemis, although it is clear he did not agree with LeConte’s (1854) synonymy stating: “Le genre Trichocnemis de M. J. L. Le Conte, établi primitivement sur la femelle d’une espèce (3) de Californie, a étè reconnu, plus tard, par se savant entomologiste, comme devant rentrer dans celui-ci. Cette femelle, que j’ai sous les yeux, diffère notablement, sous le rapport du *facies*, de celle de *faber*, et a celui d’une Macrotoma; son prothorax est multiépineux sur les côtés et les épines sont longues et irrégulières. D’après la description qu’en donne M. J. L. Le Comte, le mâle différerait également, d’une manière sensible, de celui de l’espèce européenne.”

LeConte (1869) was the first author to attribute subgeneric status to Trichocnemis, when he listed “Ergates (Trichocnemis) spiculatus Lec.,” but did not offer an explanation of this new status. Casey (1890) maintained Trichocnemis as a subgenus of Ergates based on the length of the antennae, anterior legs, and denticulation of the sides of the prothorax, stating: “It seems proper therefore that the name Trichocnemis Lec. should be preserved, if not with full generic value, at least as a subgenus.”

Lameere (1901) considered Trichocnemis different from Ergates (“genre très distinct”), and included both in the tribe “Aegosomites” and subtribe “Callipogonines.” Lameere (1904) assigned Ergates to “Callipogonines,” and divided it into three subgenera: Ergates (Ergates); Ergates (Trichocnemis); and Ergates (Callergates) Lameere, 1904. Ergates (Callergates) is currently considered a different genus (Jeniš 2001, 2008).

Casey (1912) again considered Ergates and Trichocnemis as distinct taxa, stating: “This genus is distinct from Ergates and should be restored. The last joint of the maxillary palpi in Ergates is oval and more narrowly truncate at apex, while in Trichocnemis it is of a wholly different form, being broadly obtriangular, the sides straight and widely flaring from base to the truncate apex. In the former there is a prominent lateral spiniform tooth at basal third of the prothorax, wanting in the latter, and there are numerous other incongruities. The two genera are related tribally but are unquestionably distinct.” While this diagnosis points out many of the differences between the two genera, several other characters previously mentioned (i.e. antennal length and ratios) were omitted. In addition, Casey (op. cit.) did not indicate a tribal assignment for either of these genera. Nevertheless, taken as a whole, the characters enumerated by Casey (1890, 1912) indicate substantial differences between Ergates and Trichocnemis.

Nishio (1956) pointed out that “the three species of Ergates strongly differ from each other in their morphology” and followed Lameere (1904) in maintaining each species in a different subgenus. Nishio (op. cit.) also compared the male genitalia of the three species, and stated (translated): “The male genitalia of gaillardoti and spiculatus are similar to each other and probably suggest that they are closely related…”. In addition, Nishio (op. cit.) hypothesized the phylogenetic relationship among the three taxa, stating that Callergates gailardoti is the most ancestral species of “Ergates,” Trichocnemis spiculatus is sister to it, and Ergates faber is the most derived species. Moreover, Nishio (op. cit.) cites Plavilstshikov (1936) as stating that “spiculatus” differs from the remaining species (Ergates faber and Callergates gaillardoti) and should be classified in a different genus.

Linsley (1962) synonymyzed Trichocnemis under Ergates, and assigned it to the tribe Ergatini, stating: “I agree with LeConte and Horn that the characters do not warrant the generic recognition of Trichocnemis and that the phylogenetic unity of the group is better indicated by including all four species in Ergates”. In placing Ergates in the tribe Ergatini, Linsley (op. cit.) apparently ignored the name “Callipogonitae” used by Thomson (1861), and gave “Ergatites,” used by Fairmaire (1864), priority over the names that appear in Lameere (1904, 1912, 1919): “Callipogonines”; Callipogonini. There seems little doubt that Linsley was aware of the name “Callipogonides” in Lacordaire (1869), and probably incorrectly attributed this taxon to him. This would explain why Lisnley (op.cit.) did not use Thomson’s name for Ergates and Callipogon in the same tribe: “This tribe is represented in America by two genera, Callipogon and Ergates”. Therefore, to Linsley, Callipogonini was equal to Ergatini, and not a different group as considered formerly and by some contemporary authors.

In his work on the Cerambycidae of France, Villiers (1978) considered the three subgenera erected by Lameere (1904) as distinct genera, stating: “Trichocnemis J. LeConte et Callergates Lameere no sont pas des sous-genres d’Ergates, mais des genres bien individualisés”. Villiers (op. cit.), used the tribal name Ergatini, although in a different sense from that employed by Linsley (op. cit.); to him, Callipogonini sensu Lameere included more than one tribe, while Linsley (op. cit.) used Ergatini only as a name with priority over Callipogonini.

The revalidation of Trichocnemis by Villiers (op.cit.) as a separate genus remained unnoticed by many contemporary authors possibly because it was published as a part of a regional faunal account. Nevertheless, subsequent checklists of Western Hemisphere Cerambycidae (Chemsak and Linsley 1982, Monné and Giesbert 1994) unintentionally maintained the synonymy of Trichocnemis with Ergates.

Although the tribal classification of Ergates and Callipogon Audinet-Serville, 1832, is beyond the scope of this paper, it is interesting to note Švácha (1987): “I would like to point out that it is undoubtedly incorrect to classify the genera Ergates and Callipogon in the same tribe, whatever its name may be.” Unfortunately, Švácha did not enumerate the characters he used to base his opinion. Today, researchers of the Neotropical and Nearctic cerambycid fauna use Callipogonini sensu Lameere (1904), while those that work with the fauna of Palearctic, Ethiopian, Oriental, and Australian zoogeographic provinces (with some exceptions), do not agree and use more than one tribe to allocate the genera included by Lameere in Callipogonini.

A partial bibliography of Trichocnemis is listed below, including many citations of the generic name Ergates which actually refer to Trichocnemis (Monné 2006).

### Trichocnemis

LeConte, 1851

Trichocnemis LeConte 1851: 110 (type species: Trichocnemis spiculatus LeConte, 1851, original designation); Melsheimer 1853: 100; White 1853: 35 (syn. under Macrotoma); LeConte 1854: 218 (synonymy under Ergates); Thomson 1861: 315 (involuntary revalidation); 1864: 298; Lacordaire 1869: 95 (involuntary synonymy); LeConte and Horn 1883: 271 (synonymy); Casey 1912 (revalidation; new status); Linsley 1962: 24 (synonymy); Villiers 1978: 55 (revalidation); Monné 1995: 15 (cat.; involuntary synonymy).
Ergates  (Trichocnemis) LeConte 1869: 371 (reval.; new status); Casey 1890: 490 (revalidation); 1891: 20; Lameere 1904: 46; 1913: 46 (cat.; reversion of status); Lameere 1919: 81; Blackwelder 1946: 553 (cat.); Nishio 1956: 68.
Ergates Horn 1891: 41; Leng 1884: 8; Arnett 1962 855; 874:; Chemsak 1996: 84; Monné 2006: 37 (cat.; part); Monné and Hovore 2006: 10 (cat.; part).
Macrotoma White 1853: 35 (part).

#### Redescription.

**Figures 7–12.**
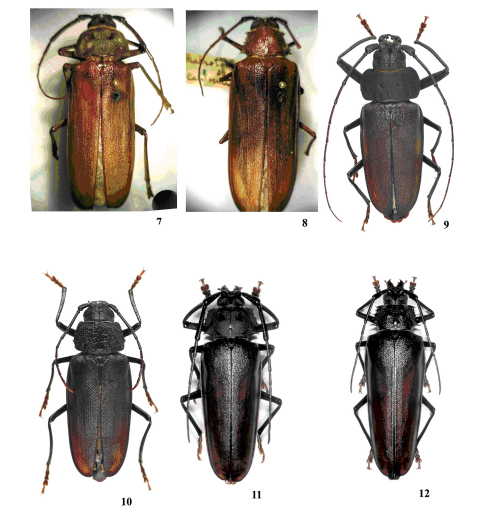
**7** Macrotoma californica, syntype 2 **8** Macrotoma spiculigera, holotype female **9** Ergates faber, male **10** idem, female **11** Callergates gaillardoti, male **12** idem, female.

 Body large, elongate, integument light brown to dark-brown; in general, elytra lighter than the head and the pronotum. Male (Figs 1,3, 5–7). Head proportionally small; coronal suture clearly surpasses the posterior edge of the eyes; dorsal surface coarsely punctate; pilosity short and scattered. Area behind the eyes confluent punctate; pilosity short and clearly more abundant than in dorsal surface of the head. Antennal tubercles moderately prominent; apex rounded. Eyes small, not as long as scape in lateral view, and lower lobe narrower than scape at its widest point; dorsal interocular space equal or just narrower than twice the width of one upper eye lobe. Hypostomal area depressed to slightly depressed, rugose-punctate. Mandibles shorter than half of the length of the head, strongly curved inwards at almost straight angle; outer surface slightly tumid at basal one-third; inner margin not tumid and not strongly separated by the punctate area. Antennae short, just attaining the apical one-third of the elytra. Scape attaining to just surpassing the posterior edge of the eye lobe. Antennomere III moderately thick, with prominent denticles on ventral and lateral surface; longer than IV-V together. Genal apex spiniform. Maxillary palps short; palpomere II longer than the others; apex of the IV securiform or barely wider than base. Prothorax strongly tumid, entirely micropunctate. Pronotum with two large, deep and subtriangular antero-medial depressions; three punctiform, small, shallow to moderate, lateral antero-medial depressions, arranged diagonally; five punctiform, small, shallow to moderate depressions, at basal area; lateral margins with spines clearly present, longer at anterior and posterior angles; lateral angles rounded; pilosity very short, very scattered (disc almost glabrous), longer and more dense laterally or close to the posterior and anterior angles. Prosternum with short and very scattered pilosity. Prosternal process wide; apex rounded; lateral margins and apical one-third with long dense pilosity. Meso-, metasternum, and metepisternum densely pilose. Elytra rugose-punctate, circum-scutellar area mostly punctate; each elytron with at least two clear carinae; sutural apex with short spine or inermis. Coxae abundantly pilose. Femora with short pilosity, becoming more dense ventrally, mainly at meso- and metafemora; profemora slightly rugose. Protibiae moderately short and thick. Protarsomere I short and wide. Urosternites pilose, mainly laterally. Parameres (lateral lobes) of the tegmen elongated, clearly narrowed, thickened, and carinate at apical half (subcylindrical).

Female (Figs 2, 4, 8). Differing from male in the following manner: antennae reaching or just surpassing middle of the elytra; scape shorter, just attaining the posterior edge of the eyes; antennomere III thinner, lacking denticles; curvature inwards at apex of the mandible at an obtuse angle; prothorax much less tumid; pronotum rugoso-punctate, strongly convex; with callosities in place of the depressions of the antero-medial and basal areas found in males, and without depressions at lateral of the antero-medial areas; lateral margins with larger and more spines (usually, the spines are bifid or trifid at apex); lateral angles clearly acute; posterior angles rounded; proepisterna coarse punctate; proepimera nearly flat; profemora laterally flattened.

#### Diagnosis.

Trichocnemis differs from Ergates (Figs 9, 10) in the following manner: head proportionally small (0.6 times greatest width of pronotum in males); mandibles not strongly tumid at basal one-third of the outer surface; inner margin of the mandible not tumid and weakly separated by a punctate furrow; antennae of males do not reach the elytral apex; scape of the males reaches or surpasses the posterior edge of the eyes; antennomere III in males clearly thicker, with denticles, longer than IV and V together; antennomere III in females longer than IV and V together, attaining or almost attaining the base of the prothorax; pronotum distinctly tumid, mainly laterally, with deep and well marked depressions at disc; proepisternum, proepimerum, and prosternum (mainly close to the head) strongly tumid; lateral margins of the pronotum with at least some spines in both sexes; anterior angles of pronotum spinose in both sexes; lateral angle of the pronotum of the males not marked; profemora of males slightly rugose; elytra rugoso-punctate, with clear carinae; protibiae of males moderately short and thick; protarsomere I short and wide in both sexes; parameres of the tegmen elongated, clearly narrowed, thickened, and carinate at apical half.

In Ergates, the head is proportionally large (0.6 times greatest width of pronotum in males); mandibles strongly tumid at basal one-third of the outer surface, mainly in males; inner margin of the mandible tumid and strongly separated by a punctate furrow; antennae of males attain or surpass the elytral apex; scape of males not attaining posterior edge of eyes; antennomere III of the males clearly thinner, without denticles, and as long as IV-V together; antennomere III of the females does not attain the base of the prothorax, as long as IV-V together; pronotum not tumid, with callosities in place of the punctate depressions found in Trichocnemis; proepisternum and proepimerum not tumid; prosternum not tumid near head; lateral margins of the pronotum crenulated in both sexes; anterior angles of the pronotum wide and rounded in both sexes; lateral angle of the pronotum with prominent spines in both sexes (lateral angles acute in males); profemora of males strongly rugose; elytra coarse and densely punctate, with feeble carinae; protibiae of the males long and narrow; protarsomere I long and narrow in both sexes; parameres of the tegmen short, not narrowed after middle, somewhat concave, thickened only at outer lateral and apical one-third.

Trichocnemis differs from Callergates (Figs 11, 12) as follows: eyes not large; prothorax with distinct lateral declivities; genitalia of male shorter, with apex of the parameres of the tegmen thickened at apical half, and the median lobe enlarged at base and distinctly convergent to the apex. In Callergates the eyes are large, the prothorax lacks lateral declivities, the genitalia of the male is longer, with the apex of the parameres of the tegmen not thickened at apical half, and the median lobe is distinct narrower at base and slightly convergent to the apex. Additionally, the protibia in males are similar to Ergates.

#### Key to the genera and species of Callergates, Ergates, and Trichocnemis

.

**Table:** 

1	Antennae surpassing middle of elytra; pronotum with distinct small, shining, impunctate areas contrasting with the remainder of the surface. Males	2
–	Antennae reaching, at most, middle of elytra; pronotum without distinct small shining, impunctate areas contrasting with the remainder of the surface. Females	5
2(1)	Apex of antennal scape not surpassing posterior margin of lower eye lobe; antennomere III slender, lacking denticles; prolegs longer than meso- and metalegs. Europe, NW Africa	Ergates faber (Linnaeus, 1761) (Fig. 9)
–	Apex of antennal scape surpassing posterior margin of lower eye lobe; antennomere III distinctly thickened, with numerous denticles; prolegs not longer than meso- and metalegs	3
3(2)	Scape distinctly surpassing the anterior margin of pronotum; antennomere III not distinctly longer than IV and V together; metasternum with a deep, somewhat small depression close to the mesocoxae. Europe, Asia Minor	Callergates gaillardoti (Chevrolat, 1854) (Fig. 11)
–	Scape reaching, at most, the anterior margin of pronotum; antennomere III distinctly longer than IV-V together; metasternum without deep depression close to the mesocoxae	4
4(3)	Inner apical angles of elytra spined, elytra either uniformly dark brown (California) or with light brown maculae (western USA); lateral spines of pronotum of differing lengths. United States and Mexico (Baja California)	Trichocnemis spiculatus LeConte, 1851 (Figs 3, 5–7)
–	Inner apical angles of elytra rounded, elytra uniformly light brown, contrasting with pronotum; lateral spines of pronotum generally of equal length. United States (Sierra Nevada and Coast Range mountains of California)	Trichocnemis pauper Linsley, 1957 (Fig. 1)
5(1)	Distance between upper ocular lobes larger than twice the width of a single lobe; pronotum not spined laterally	Ergates faber (Linnaeus, 1767) (Fig. 10)
–	Distance between upper ocular lobes smaller than twice the width of a lobe; pronotum spined laterally	6
6(5)	Apex of antennal scape distinctly surpassing posterior margin of lower eye lobe; antennomere III as long as IV-V together or barely longer	Callergates gaillardoti (Chevrolat, 1854) (Fig. 12)
–	Apex of antennal scape not or just surpassing posterior margin of lower eye lobe; antennomere III distinctly longer than IV-V together	7
7(6)	Spines of lateral margins of pronotum as long as those at anterior and lateral angles; sutural angle of elytra unarmed	Trichocnemis pauper Linsley, 1957 (Fig. 2)
–	Spines of lateral margins of pronotum shorter than those at anterior and lateral angles; sutural angle of elytra with short spine	Trichocnemis spiculatus LeConte, 1851 (Figs 4, 8)

## Conclusions

Our analysis of these taxa, which corroborates that of Villiers (1978) and in part, those of Lameere (1904) and Nishio (1956), supports recognizing Trichocnemis and Ergates as distinct genera. Additionally, the fact that both species of North American Trichocnemis share several distinct characters not present in Ergates or Callergates further supports this hypothesis.

## Summary of taxonomic changes

Trichocnemis spiculatus spiculatus LeConte, 1851 (originally described as Trichocnemis spiculatus LeConte, 1851); Trichocnemis spiculatus neomexicanus (Casey, 1890) (originally described as Ergates (Trichocnemis) neomexicanus Casey, 1890), comb. n.; Trichocnemis pauper (Linsley, 1957) (originally described as Ergates pauper Linsley, 1957), comb. n.

## Synonyms of Trichocnemis spiculatus LeConte, 1851

White (1853) described two species from North America (California) that were later synonymyzed with Trichocnemis spiculatus by Lameere (1904): Macrotoma californica and Macrotoma spiculigera. White’s original description leaves some doubt as to the identity of the species involved. For example, in the description of Macrotoma spiculigera, he stated: “Elytra coriaceous, vermiculated, with three indistinct costae”. Similarly, some details of the description of Macrotoma californica might encompass that of Trichocnemis pauper. Since White probably did not examine the types of these species (frequently he indicated when he did), and his original descriptions do not provide enough detail to diagnose them among other Trichocnemis, primarily Trichocnemis pauper, we examined photos of the types, provided by S. Shute (BMNH).

The syntype male of Macrotoma californica (Figs 6, 8) and the holotype female of Macrotoma spiculigera (Fig. 8), are in fact Macrotoma spiculatus, as suspected by even White (op.cit.) himself: “Trichocnemis spiculatus, Leconte, *Journ. Acad. Nat. Sc. Phil.* n. s. ii 110?”, and “It is possible that this may be the female of the Macrotoma Californica”. Photos of the holotype (Fig. 8) also clearly show three distinct carinae on each elytron, rather than three on the elytra. According to S. Shute (personal communication) the types have the following labels:

Macrotoma californica: Syntype 1 (Fig. 6): White H/W determination label (specimen also bears small circular white H/W  BM(HN) registration label  upper surface reads California, reverse [18] 48 . 135  (the register states that this specimen was purchased from Hartweg);

Syntype 2 (Fig. 7): no labels other than blue BM(NH) syntype label;

Macrotoma spiculigera (Fig. 8): White H/W label. The reverse of this label has Hermerius struck out in black ink and California written below. The generic name is in the large script of White and must have been the original label. This specimen also has a small white circular registration as for Macrotoma calfornica  [18]48 . 135 plus BM(NH) red type label.

## Supplementary Material

XML Treatment for Trichocnemis
